# Different Effects of Soil Fertilization on Bacterial Community Composition in the *Penicillium canescens* Hyphosphere and in Bulk Soil

**DOI:** 10.1128/AEM.02969-19

**Published:** 2020-05-05

**Authors:** Yuan Zhang, Xiuli Hao, Adriana M. Garcia-Lemos, Inês Nunes, Mette H. Nicolaisen, Ole Nybroe

**Affiliations:** a Section for Microbial Ecology and Biotechnology, Department of Plant and Environmental Sciences, University of Copenhagen, Frederiksberg C, Denmark; b State Key Laboratory of Agricultural Microbiology, Key Laboratory of Arable Land Conservation (Middle and Lower Reaches of Yangtze River), Ministry of Agriculture, College of Resources and Environment, Huazhong Agricultural University, Wuhan, China; c Microbiomics and Microbe Discovery, Novozymes A/S, Kongens Lyngby, Denmark; Nanjing Agricultural University

**Keywords:** soil fungi, baiting microcosm, phosphorus and nitrogen cycling genes, long-term soil fertilization, bacterial communities, filamentous fungi, soil microbiology

## Abstract

This study investigated the effects of long-term soil fertilization on the composition and potential for phosphorus (P) and nitrogen (N) cycling of bacterial communities associated with hyphae of the P-solubilizing fungus Penicillium canescens. Using a baiting approach, hyphosphere bacterial communities were recovered from three soils that had received long-term amendment in the field with mineral or mineral plus organic fertilizers. *P. canescens* hyphae recruited bacterial communities with a decreased diversity and an increased abundance of *Proteobacteria* relative to what was observed in soil communities. As core bacterial taxa, Delftia and Pseudomonas spp. were present in all hyphosphere samples irrespective of soil fertilization. However, the type of fertilization showed significant impacts on the diversity, composition, and distinctive taxa/operational taxonomic units (OTUs) of hyphosphere communities. The soil factors P (Olsen method), exchangeable Mg, exchangeable K, and pH were important for shaping soil and hyphosphere bacterial community compositions. An increased relative abundance of organic P metabolism genes was found in hyphosphere communities from soil that had not received P fertilizers, which could indicate P limitation near the fungal hyphae. Additionally, *P. canescens* hyphae recruited bacterial communities with a higher abundance of N fixation genes than found in soil communities, which might imply a role of hyphosphere communities for fungal N nutrition. Furthermore, the relative abundances of denitrification genes were greater in several hyphosphere communities, indicating an at least partly anoxic microenvironment with a high carbon-to-N ratio around the hyphae. In conclusion, soil fertilization legacy shapes *P. canescens* hyphosphere microbiomes and their functional potential related to P and N cycling.

**IMPORTANCE** P-solubilizing Penicillium strains are introduced as biofertilizers to agricultural soils to improve plant P nutrition. Currently, little is known about the ecology of these biofertilizers, including their interactions with other soil microorganisms. This study shows that communities dominated by *Betaproteobacteria* and *Gammaproteobacteria* colonize *P. canescens* hyphae in soil and that the compositions of these communities depend on the soil conditions. The potential of these communities for N and organic P cycling is generally higher than that of soil communities. The high potential for organic P metabolism might complement the ability of the fungus to solubilize inorganic P, and it points to the hyphosphere as a hot spot for P metabolism. Furthermore, the high potential for N fixation could indicate that *P. canescens* recruits bacteria that are able to improve its N nutrition. Hence, this community study identifies functional groups relevant for the future optimization of next-generation biofertilizer consortia for applications in soil.

## Abstract (precis)

P-solubilizing Penicillium strains are introduced as biofertilizers to agricultural soils to improve plant P nutrition. Currently, little is known about the ecology of these biofertilizers, including their interactions with other soil microorganisms. This study shows that communities dominated by *Betaproteobacteria* and *Gammaproteobacteria* colonize *P. canescens* hyphae in soil and that the compositions of these communities depend on the soil conditions. The potential of these communities for N and organic P cycling is generally higher than that of soil communities. The high potential for organic P metabolism might complement the ability of the fungus to solubilize inorganic P, and it points to the hyphosphere as a hot spot for P metabolism. Furthermore, the high potential for N fixation could indicate that *P. canescens* recruits bacteria that are able to improve its N nutrition. Hence, this community study identifies functional groups relevant for the future optimization of next-generation biofertilizer consortia for applications in soil.

## INTRODUCTION

Fungal hyphae provide a unique niche for bacterial colonization in which bacteria benefit from their fungal partner by obtaining suitable carbon (C) or energy sources from fungal exudates (1, 2). In return, the specific bacterial communities recruited by fungal hyphae may help the host fungi by accessing recalcitrant forms of soil nutrients and hence meet the host’s nutrient requirements (3). Fungi are key components in soil environments that drive important ecosystem functions (4), and fungal hyphae may serve as hot spots for nutrient cycling. For example, Zhang et al. (2) observed that the bacterial communities attached to surfaces of hyphae from arbuscular mycorrhizal fungi (AMF) can produce alkaline phosphatase, a function absent from fungal hyphae. Another study revealed that association with nitrogen-fixing bacteria enabled wood-decaying fungi to overcome nitrogen (N) depletion (5). However, high competition has also been observed between fungi and their associated bacteria for soil nutrients such as phosphorus (P) (6).

Penicillium spp. are common soil fungi considered to be “key organisms” involved in soil P cycling due to their ability to solubilize inorganic P by producing organic acids (7, 8). Some *Penicillium* elite strains are used as biofertilizer inoculants in agriculture to improve crop P nutrition and increase crop yield, although with varied efficacy (9, 10). Currently, the interactions between *Penicillium* spp. and the bacteria colonizing their hyphae in soil are understudied, although these bacteria might develop important interactions with the inoculants in the soil. For comparison, hyphosphere bacteria (the term “hyphosphere bacteria” is used below for bacteria living on, or in association with, hyphae) can exert positive effects on AMF mycelial growth and mycorrhiza formation (1), while studies for Rhizoctonia spp. have revealed several bacterial isolates that inhibit fungal growth (11).

Several baiting systems have been constructed in order to study the hyphosphere bacteria of different fungi (12–14). These systems are most often based on extracted soil or rhizosphere bacteria, which are added to hyphae growing on an artificial medium. Therefore, bias may arise due to the different physiological statuses of hyphae when grown in artificial medium versus in natural soil, which might lead to the release of different exudates that can attract bacteria. Recently, a baiting soil microcosm approach was developed in which *Penicillium* hyphae are incubated in natural soil, enabling the recovery and analysis of bacterial communities associated with the hyphae under realistic soil conditions (15). The results obtained using this baiting system show that Penicillium bilaiae hyphae recruited a specific bacterial community from one investigated soil (15), and that very similar bacterial communities develop in association with hyphae of *P. canescens* and Penicillium janthinellum grown in the same soil. However, different hyphosphere communities develop when different top soils and subsoils with various characteristics are used in the baiting system (16). Nevertheless, insight into the factors that influence the composition of hyphosphere communities is still very limited.

Mineral and organic fertilizers are routinely applied to improve nutrient availability to crops and, ultimately, crop yields in agricultural systems. The impacts of fertilizer regime on the diversity and structure of microbial communities in the soil have been well studied (17–20). Mineral fertilizers have been reported to affect soil microbial community composition directly by supplying nutrients to the microorganisms or indirectly by changing the soil pH (18). For organic fertilizers, increased bacterial biomass and community diversity have been found in long-term trials fertilized with manure/organic amendments (17, 19, 20). However, it is still unknown how the fertilizer regime affects the composition of the *Penicillium* hyphosphere bacterial communities and their functional potential for cycling major nutrients such as N and P.

Hence, the objective of the current study was to determine the impacts of soil fertilization on the composition, as well as the potential for N and P cycling, of bacterial communities in the hyphosphere of the P-solubilizing *P. canescens* under close-to-natural soil conditions. It was hypothesized that both the composition and the nutrient cycling potential of hyphosphere bacterial communities are influenced by the hyphosphere and the type of soil fertilization to which they are exposed. Baiting soil microcosms were set up in which hyphae of *P. canescens* were introduced to soils that had received long-term amendment with mineral or mineral plus organic fertilizers in the field. The bacterial communities were characterized by 16S rRNA gene amplicon sequencing, and their functional potential was determined using quantitative PCR (qPCR) targeting genes involved in N and P cycling.

## RESULTS

### Hyphal viability and bacterial colonization.

**FIG 1:**
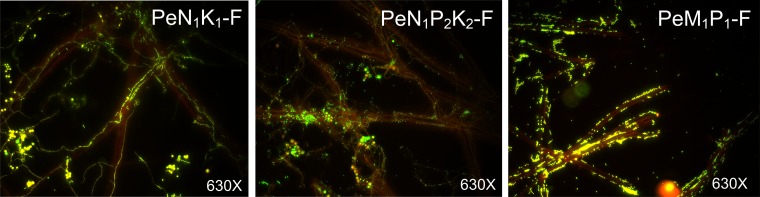
Colonization of bacteria on *P. canescens* hyphae in soil microcosms after 8 days of incubation. The *Penicillium* hyphae came from microcosms with soils that had been amended in the field over a long time with N and K (PeN_1_K_1_-F), N, P, and K (PeN_1_P_2_K_2_-F), or manure plus P (PeM_1_P_1_-F). Hyphae and bacteria were stained by SYBR green. Images were obtained by fluorescence microscopy at ×630 magnification.

Baiting microcosms were established in three soils that differed in the type of fertilizer amendments, N_1_K_1_-F, N_1_P_2_K_2_-F, and M_1_P_1_-F (Table 1). Glass coverslips colonized by *P. canescens* hyphae were recovered from N_1_K_1_-F soil microcosms and analyzed for hyphal morphology and viability at different time points (see Fig. S1A in the supplemental material). Intact hyphae were observed using calcofluor white staining (images a to d in Fig. S1A), and a mixture of hyphae with and without metabolic activity was observed by FUN1 staining, yielding green fluorescence from inactive hyphae and spores versus red-orange fluorescence from active hyphae and spores (images e to h and i to p in Fig. S1A). At day 0, a large number of active spores and a small number of active hyphae were observed, while at days 1, 4, and 8, old and active hyphae were not observed, whereas germinating spores and young active hyphae were abundant. After 8 days of incubation, the *P. canescens* hyphae from microcosms established in all three soils were colonized by a large number of individual bacteria and/or bacterial aggregates, as shown by SYBR green staining (Fig. 1). In contrast, the negative-control glass coverslips (slides without *P. canescens* hyphae) from the three microcosms showed very few bacteria attached onto the glass (Fig. S1B).

**TABLE 1:** General properties of soils used for establishing baiting microcosms and specifications of long-term nutrient amendments in the field

Soil sample	Soil origin^*a*^	Fertilizer amendment(s)	pH	Olsen P (mg kg^−1^)	Content (mg kg^−1^) of exchangeable:		Total content:			
					K	Mg	C (g kg^−1^)	N (g kg^−1^)	P (mg kg^−1^)	K (mg kg^−1^)
N_1_K_1_-F	LTNDT N_1_K_1_ field	Mineral	5.2	7.5	134.3	66.0	12.3	1.65	355	15,211
N_1_P_2_K_2_-F	LTNDT N_1_P_2_K_2_ field	Mineral	5.1	16.3	219.8	57.3	12.8	1.65	452	15,452
M_1_P_1_-F	LTNDT M_1_P_1_ field	Organic and mineral	5.8	17.0	119.8	89.5	13.9	1.70	423	14,982

a LTNDT, long-term nutrient depletion trial.

### Diversity and structure of soil and hyphosphere bacterial communities.

**FIG 2:**
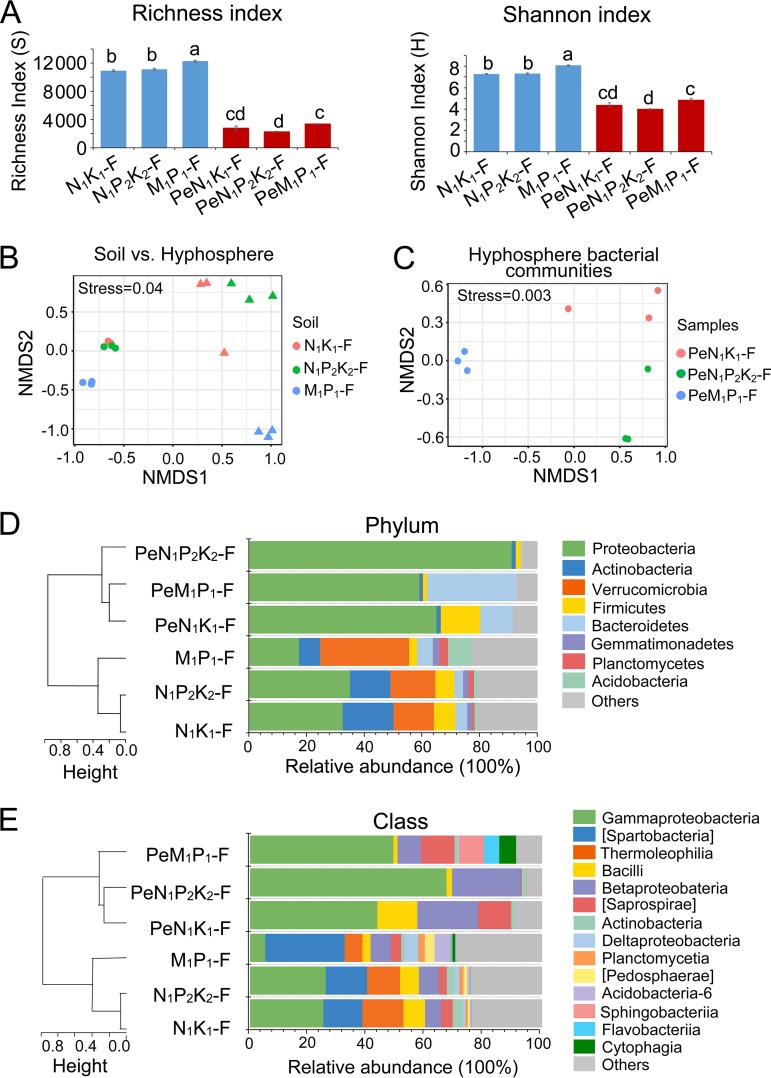
Diversity, structure, and composition of soil and hyphosphere bacterial communities. The soil communities came from soils that had been amended in the field over a long period with N and K (N_1_K_1_-F), with N, P, and K (N_1_P_2_K_2_-F), or with manure plus P (M_1_P_1_-F). Hyphosphere communities recruited from these soils are referred to as PeN_1_K_1_-F, PeN_1_P_2_K_2_-F, and PeM_1_P_1_-F, respectively. (A) Alpha-diversity indices (richness and Shannon index) calculated from 16S rRNA gene sequence data at the OTU level (blue, soil samples; red, hyphosphere samples). Error bars represent the standard error of the results from three replicates. The statistical significance of differences was analyzed for all samples using one-way ANOVA, followed by the Duncan test (*P* < 0.05), and is indicated by lowercase letters; different letters indicate significant differences. (B) NMDS plots for soil and hyphosphere bacterial community structures based on weighted UniFrac distance. Circles indicate soil communities, while triangles indicate hyphosphere communities. (C) NMDS plots for hyphosphere bacterial community structure based on weighted UniFrac distance. (D and E) Community composition of soil and hyphosphere samples at the phylum (D) and class (E) levels. The stacked bar charts depict the mean relative abundance of the taxa present in each sample. “Others” represents bacterial taxa with mean relative abundances below 2% and unassigned bacterial taxa. All samples were clustered based on the Bray-Curtis dissimilarity of bacterial relative abundance data.

Across treatments, soil bacterial communities had significantly higher diversity indices (richness and Shannon) than did the hyphosphere communities (*P* < 0.01) (Fig. 2A). The community from the soil receiving long-term manure plus P amendment (M_1_P_1_-F) had significantly higher diversity indices than did other soil communities (*P* < 0.05). Comparisons of hyphosphere communities exhibited comparable results because the diversity indices were significantly higher for the hyphosphere bacterial community recruited from the M_1_P_1_-F soil (referred to as PeM_1_P_1_-F) than for the hyphosphere community recruited from N_1_P_2_K_2_-F soil (*P* < 0.05). However, the hyphosphere community recruited from the N_1_K_1_-F soil had diversity indices similar to those recruited from the M_1_P_1_-F soil.

Nonmetric multidimensional scaling (NMDS) analysis of operational taxonomic unit (OTU) matrices, based on weighted UniFrac distances, revealed a clear separation between soil bacterial communities and hyphosphere communities (permutational multivariate analysis of variance [PERMANOVA], *F *= 24.338, *R* = 0.6, *P* < 0.001) (Fig. 2B). Furthermore, long-term soil fertilization had strong and significant effects on the structures of both soil (PERMANOVA, *F* = 10,844, *R* = 0.8, *P* < 0.005) and hyphosphere (PERMANOVA, *F* = 47,629, *R* = 0.9, *P* < 0.005) bacterial communities, in particular for communities from M_1_P_1_-F and PeM_1_P_1_-F, which clustered separately from the other communities (Fig. 2C and S2).

### Composition of soil and hyphosphere bacterial communities.

Consistent with the diversity and NMDS analyses, hierarchical clustering analysis highlighted the dissimilarity between soil and hyphosphere communities at both the phylum and class levels (Fig. 2D and E). Within the soil community cluster, the M_1_P_1_-F community was separated from the other soil communities and was characterized by a dominance of *Verrucomicrobia* (31%), while the most-abundant phylum in the other two soil communities was *Proteobacteria*, with a relative abundance ranging from 30% to 35%. Moreover, the relative abundances of *Firmicutes* and *Actinobacteria* were significantly lower in M_1_P_1_-F (*P* < 0.05).

Compared with the corresponding soil communities, hyphosphere communities presented significantly higher relative abundances of *Proteobacteria* (*P* < 0.01) and lower abundances of *Firmicutes* (*P* < 0.05, with the exception of PeN_1_K_1_-F) (Table S1). Furthermore, PeM_1_P_1_-F had a significantly higher abundance of the phylum *Bacteroidetes* than did M_1_P_1_-F (*P* < 0.01) (Table S1). For *Proteobacteria*, the class *Gammaproteobacteria* had a relative abundance above 45% in all hyphosphere communities (Fig. 2E). The relative abundance of the class *Betaproteobacteria* varied from over 20% in hyphosphere communities retrieved from field soil with NPK and NK fertilizer amendments (PeN_1_P_2_K_2_-F and PeN_1_K_1_-F, respectively) to 8% in hyphosphere samples from field soil receiving manure plus P fertilizer (PeM_1_P_1_-F).

**FIG 3:**
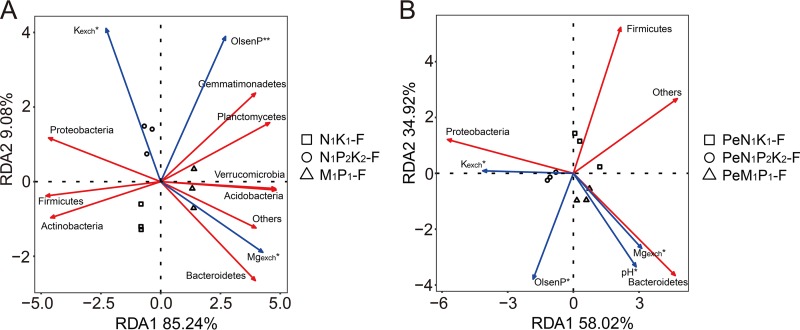
(A and B) Redundancy analysis (RDA) of soil properties and the relative abundance of bacterial taxa (phylum level) in soil communities (A) and in hyphosphere bacterial communities (B). The soil communities came from soils that had been amended in the field over a long period with N and K (N_1_K_1_-F), with N, P, and K (N_1_P_2_K_2_-F), or with manure plus P (M_1_P_1_-F). Hyphosphere communities recruited from these soils are referred to as PeN_1_K_1_-F, PeN_1_P_2_K_2_-F, and PeM_1_P_1_-F, respectively. The significance of factors was determined by the Monte Carlo permutation test (999 permutations). Asterisks indicate the level of significance at *P* values of <0.05 (*) and <0.01 (**). The soil factors analyzed were as follows: C/N, carbon-to-nitrogen ratio; K_exch_, exchangeable potassium; Mg_exch_, exchangeable magnesium; TK, total potassium; and TP, total phosphorus. Phyla with relative abundances of >2% are shown as vectors in the RDA representations. Others include bacterial phyla with a mean relative abundance of <2% and unassigned bacterial taxa.

The effects of soil factors on the relative abundances of phyla in the soil samples were determined by redundancy analysis (RDA), where the first two axes of the RDA explained 85% and 9% of the total variation, respectively (Fig. 3A). The first axis separated soil with mineral fertilizer amendments (N_1_K_1_-F and N_1_P_2_K_2_-F) from soil with the organic fertilizer amendment (M_1_P_1_-F), while the second axis separated the N_1_P_2_K_2_-F soil from the other soils. Olsen P, exchangeable K, and exchangeable Mg were significant factors affecting the relative abundances of phyla in these soils (*P* < 0.05). A significantly positive correlation was observed between Olsen P and the relative abundances of *Gemmatimonadetes* and *Planctomycetes* (Pearson correlation coefficient, *r* = 0.84 and 0.75, respectively, *P* < 0.05). *Bacteroidetes* showed a significantly negative correlation with exchangeable K (*r* = −0.86, *P* < 0.05). Exchangeable Mg was significantly and positively correlated with the relative abundances of *Verrucomicrobia* (*r* = 0.89), *Bacteroidetes* (*r* = 0.89), *Planctomycetes* (*r* = 0.69), and *Acidobacteria* (*r* = 0.88) (*P* < 0.05) but was negatively correlated with *Proteobacteria* (*r* = −0.93), *Actinobacteria* (*r* = −0.74), and *Firmicutes* (*r* = −0.84) (*P* < 0.05).

An RDA was conducted for hyphosphere samples, with the analysis relating bulk soil factors to the recruitment of bacterial taxa to the hyphae (Fig. 3B). For the hyphosphere samples, the first two axes of the RDA explained 58% and 35% of the total variation, respectively. Here, the first axis seemed to separate the N_1_P_2_K_2_-F soil from the other soils. Soil pH, Olsen P, exchangeable K, and exchangeable Mg were significant factors affecting the relative abundances of phyla associated with the hyphae (*P* < 0.05) (Fig. 3B). Across hyphosphere samples, the relative abundance of *Bacteroidetes* was significantly and positively correlated with pH (*r* = 0.87, *P* < 0.05) and exchangeable Mg (*r* = 0.82, *P* < 0.05) and negatively correlated with exchangeable K (*r* = −0.68, *P* < 0.05). In contrast, the relative abundance of *Proteobacteria* was negatively correlated with pH (*r* = −0.70, *P* < 0.05) and exchangeable Mg (*r* = −0.71, *P* < 0.05) but positively correlated with exchangeable K (*r* = 0.84, *P* < 0.05). The relative abundance of *Firmicutes* showed a significantly negative relationship with Olsen P (*r* = −0.96, *P* < 0.05).

This study then investigated whether the changes in soil and hyphosphere communities caused by fertilizer amendments were correlated, and a Mantel test was conducted to test the hypothesis that distance matrices for soil communities are not linearly related to the distance matrices for hyphosphere communities. When the fertilizer amendments were taken into account, they were found to be good predictors of the degree of similarity between soil and hyphosphere communities (*r*_Mantel_ = 0.84; *P* < 0.001, weighted UniFrac).

**FIG 4:**
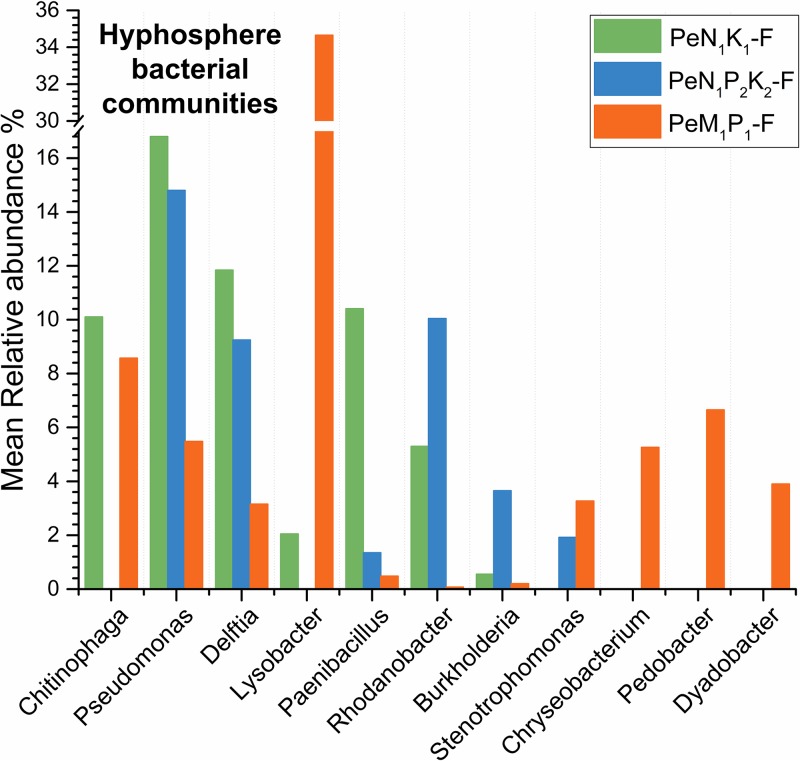
Mean relative abundances of the 11 most-abundant bacterial genera in hyphosphere communities of *P. canescens* across the microcosms. The hyphosphere communities were recruited from soils that had been amended in the field over a long time with N and K (PeN_1_K_1_-F), N, P, and K (PeN_1_P_2_K_2_-F), or with manure plus P (PeM_1_P_1_-F). The core genera were defined as the bacterial genera with a mean relative abundance above 2% in all hyphosphere communities, irrespective of soil background.

A linear discriminant analysis (LEfSe) of hyphosphere samples identified distinctive bacterial taxa at different levels depending on different soil fertilizer treatments (*P* < 0.05; Fig. S3 and Table S2). Hence, *Firmicutes* were distinctive in PeN_1_K_1_-F, *Proteobacteria* were distinctive in PeN_1_P_2_K_2_-F, and *Bacteroidetes* were distinctive in PeM_1_P_1_-F. To identify the core hyphosphere microbiome, the relative abundances of the 11 most-abundant bacterial genera were compared across all hyphosphere samples (Fig. 4). According to this analysis, the genera *Delftia* and *Pseudomonas* were core bacterial taxa of the *P. canescens* hyphosphere bacterial communities, as they were present at relative abundances of >2% in all hyphosphere samples irrespective of soil fertilization (Fig. 4).

**FIG 5:**
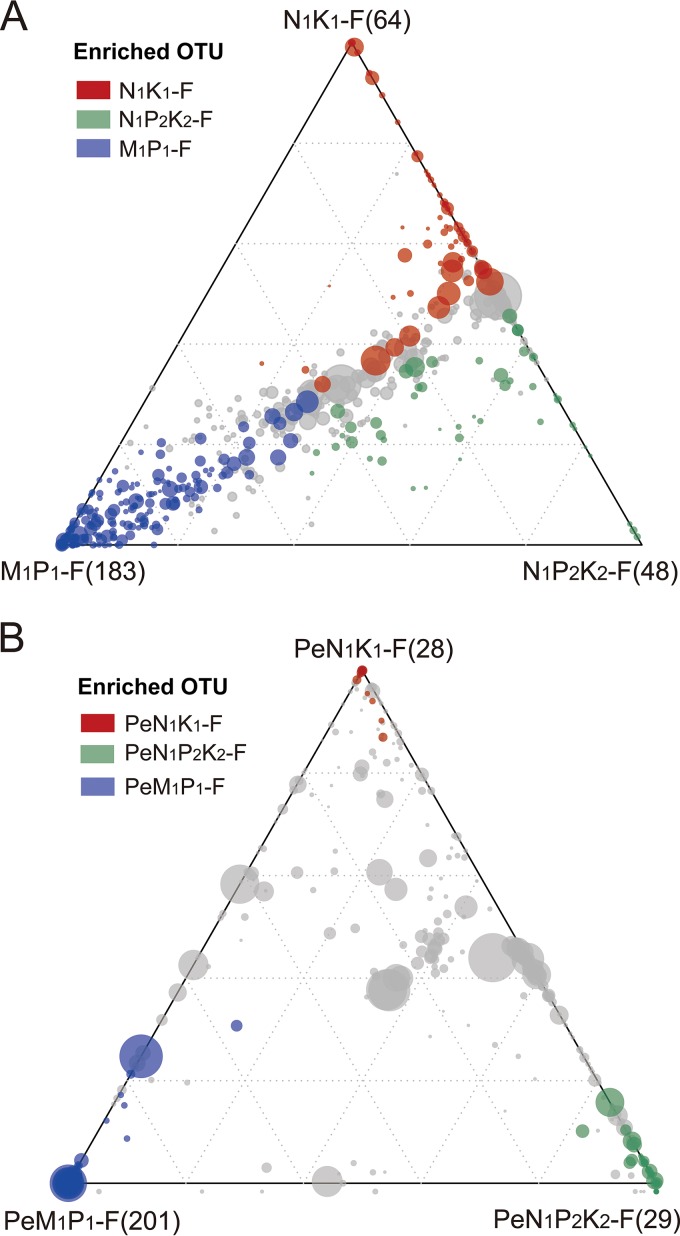
Distinct distributions of enriched OTUs in soil and hyphosphere samples. Soil communities came from soils that had been amended for a long time in the field with N and K (N_1_K_1_-F), with N, P, and K (N_1_P_2_K_2_-F), or with manure plus P (M_1_P_1_-F). Hyphosphere communities recruited from these soils are referred to as PeN_1_K_1_-F, PeN_1_P_2_K_2_-F, and PeM_1_P_1_-F, respectively. (A) OTUs specifically enriched in N_1_K_1_-F, N_1_P_2_K_2_-F, and M_1_P_1_-F. (B) OTUs specifically enriched in PeN_1_K_1_-F, PeN_1_P_2_K_2_-F, and PeM_1_P_1_-F. Ternary plots depict OTUs (>0.5‰) with respect to each compartment, where each circle corresponds to an enriched OTU. The size of each circle represents its average relative abundance across three compartments, and its position is determined by the relative abundances regarding each indicated compartment. The colored circles mark OTUs that are enriched in one compartment compared with the other compartments. OTUs that are not significantly enriched in a specific compartment are indicated by gray circles.

A comparison of the communities in N_1_K_1_-F, N_1_P_2_K_2_-F, and M_1_P_1_-F soils by ternary plot analysis showed that each soil enriched specific OTUs. In particular, the M_1_P_1_-F soil community contained a large number of specifically enriched OTUs (183 blue circles, Fig. 5A and S6). Fewer OTUs were specifically enriched in N_1_K_1_-F (64 red circles) and N_1_P_2_K_2_-F (48 green circles) (Fig. 5A, S4, and S5). The comparison of the corresponding hyphosphere communities identified 201, 28, and 29 enriched OTUs in PeM_1_P_1_-F (blue circles), PeN_1_K_1_-F (red circles), and PeN_1_P_2_K_2_-F (green circles, Fig. 5B and S7 to S9), respectively. Notably, these enriched OTUs were hardly shared between the different hyphosphere communities.

### Distribution and abundance of functional genes related to P and N cycling in soil and hyphosphere communities.

**FIG 6:**
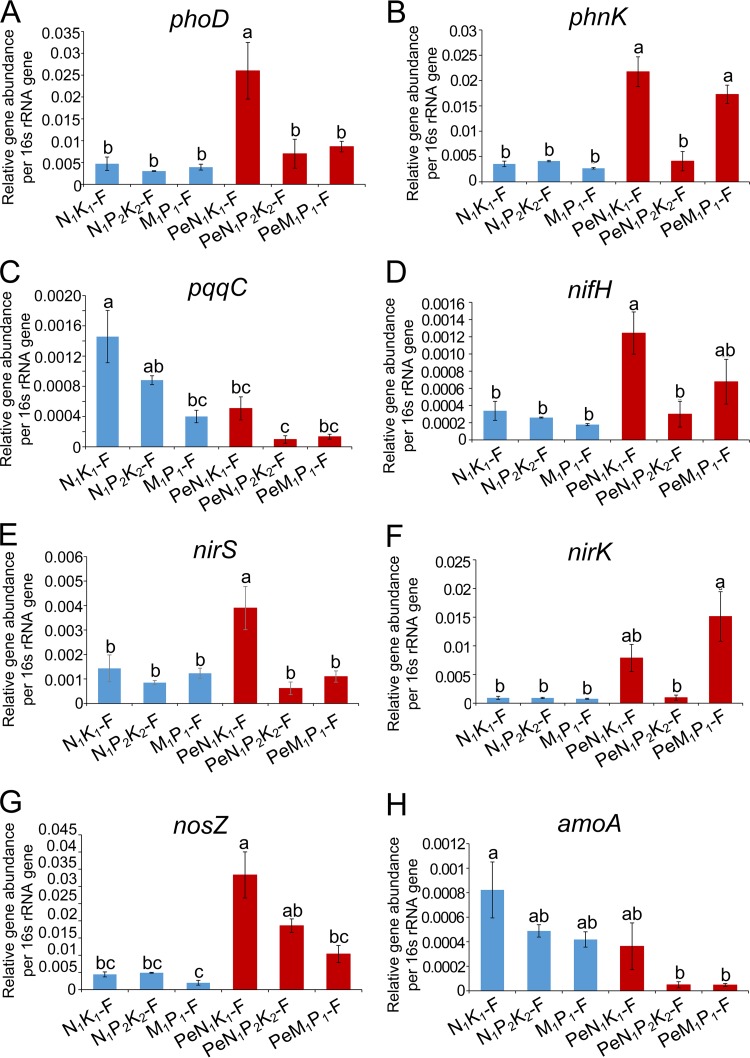
Distribution and relative abundances of functional genes involved in phosphorus and nitrogen cycling in soil and hyphosphere communities. The soil communities came from soils that had been amended in the field over a long period with N and K (N_1_K_1_-F), with N, P, and K (N_1_P_2_K_2_-F), or with manure plus P (M_1_P_1_-F). Hyphosphere communities recruited from these soils are referred to as PeN_1_K_1_-F, PeN_1_P_2_K_2_-F, and PeM_1_P_1_-F, respectively. The relative abundances of genes were calculated by the ratio of the absolute copy numbers of genes of interest to the 16S rRNA gene. The average relative abundances of the target genes in each sample are presented, and error bars represent the standard error of the results from three replicates. Significant differences were analyzed for soil samples and hyphosphere samples using one-way ANOVA, followed by Tukey’s HSD test (*P* < 0.05) and are indicated by lowercase letters; different letters indicate significant differences.

Relative abundances were investigated for functional genes related to P and N cycling for soil and hyphosphere samples (Fig. 6). The P cycling genes included *phoD*, encoding alkaline phosphatase; *phnK*, which is involved in phosphonate utilization; and *pqqC*, which is involved in pyrroloquinoline quinone biosynthesis required for inorganic P solubilization. No significant differences were observed in the relative abundances of *phoD* and *phnK* between the three soil communities. Compared to the soil communities, a significantly higher abundance (*P* < 0.05) of *phoD* was found in the PeN_1_K_1_-F hyphosphere community (Fig. 6A), while no enrichment of the *phoD* gene was found for the hyphosphere communities in the PeN_1_P_2_K_2_-F and PeM_1_P_1_-F soils. The *phnK* gene was significantly (*P* < 0.05) enriched in the PeN_1_K_1_-F and PeM_1_P_1_-F communities compared to the soil communities (Fig. 6B). For *pqqC*, the relative abundance in hyphosphere communities was generally lower than in the corresponding soil communities. This difference was significant (*P* < 0.05) for the hyphosphere communities from soils receiving inorganic fertilizers, i.e., PeN_1_K_1_-F and PeN_1_P_2_K_2_-F (Fig. 6C).

The analysis of functional genes involved in N cycling included *nifH* (nitrogenase iron protein), *nirS* (nitrite reductase), *nirK* (nitrite reductase), *nosZ* (nitrous oxide reductase), and *amoA* (ammonia monooxygenase). The relative abundances of these genes varied little between the soil communities. *nifH* had higher relative abundance in hyphosphere communities from the N_1_K_1_ and M_1_P_1_ soils than in the corresponding soil samples, although the difference was only significant (*P* < 0.05) for the comparison of N_1_K_1_-F to PeN_1_K_1_ (Fig. 6D). For hyphosphere communities recruited from the N_1_K_1_-F soil, the relative abundances of *nirS* and *nosZ* were significantly higher than those in the corresponding soil communities (*P* < 0.05) (Fig. 6E and G). Interestingly, *nirK* was significantly enriched in hyphosphere communities from the M_1_P_1_-F soil and hence showed a different pattern from that observed for *nirS* (Fig. 6F). For the *amoA* gene, the relative abundance in hyphosphere communities was lower than that in the corresponding soil communities, although these differences were not significant (Fig. 6H).

Hence, most P and N cycling genes were enriched in one or several hyphosphere communities compared to the corresponding soil communities. Furthermore, the soil fertilization regime had a stronger effect on the metabolic potential for P and N cycling in hyphosphere communities than in soil communities.

## DISCUSSION

### Colonization of hyphae in soil.

A recently developed baiting microcosm approach (15) provided an opportunity to investigate how different soil fertilizer amendments impact bacterial communities recruited by the hyphae of *P. canescens* under close-to-natural soil conditions. Bacterial colonization was observed both for old hyphae and for young hyphae developing during the incubation period from germinating spores in the soil. It could be ascertained that the bacteria recovered for molecular analysis were associated with hyphae, as very few bacteria colonized the glass coverslips that had not been inoculated with *P. canescens*, which is in agreement with previous findings for *P. bilaiae* (15).

### Effects of fertilizer regimes on bacterial community composition.

The alpha diversity of hyphosphere bacterial communities was significantly lower than that of the bacterial communities from bulk soil. This finding is consistent with previous findings for bacterial communities from mycosphere, mycelial cords (21, 22), and *Penicillium* hyphae recovered by the same baiting approach (15, 16). For soil bacterial microbiomes, the current results highlighted the influence of fertilizer type (mineral versus manures) on the structure of these communities. The bacterial community from soil with long-term manure amendment (M_1_P_1_-F) showed the highest alpha diversity and a distinct community structure, probably caused by the introduction of complex organic matter through the manure, as has been suggested by previous studies (17, 19, 20, 23).

*Proteobacteria* was the dominant phylum in all hyphosphere bacterial communities, which showed less variation in taxonomic structure than soil bacterial communities. Within the *Proteobacteria*, *Delftia* (order *Burkholderiales*) and *Pseudomonas* (order *Pseudomonadales*) were identified as core genera. These genera might play crucial roles in the provision of nutrients for the fungus, as they contain species with N-fixing and P-solubilizing activities (24–28) and are often found in association with fungi (12, 29).

The different soil fertilizer amendments had a strong and significant impact on the composition of the soil and hyphosphere bacterial communities, and RDA together with a Monte Carlo permutation test revealed that bacterial communities were influenced by the soil factors Olsen P, exchangeable K, exchangeable Mg, and pH (pH only for hyphosphere communities). For bulk-soil communities, a previous study using a wider range of soils from the long-term nutrient depletion trial showed that N inputs to soil influenced bacterial community structures more than did P or K inputs (17). The significant impact seen here for available K and Mg differed from the results from a recent microcosm study. That study showed an impact on the distribution of bacteria from mineral-weathering taxa, such as Burkholderia and Collimonas, but little effect on the overall taxonomic community structure of bacteria in forest soil (30). This discrepancy might point to a legacy effect of the repeated annual fertilizer amendments in the present study. Finally, pH has often been reported to be an important driver of soil bacterial community composition (17, 31, 32).

For hyphosphere communities, a previous study including a total of five very different topsoils and subsoils indicated that soil pH, Mg^2+^, Olsen P, and total carbon influenced bacterial communities associated with *P. canescens* hyphae (16). Hence, the current results consolidate these previous observations using a targeted fertilizer amendment approach. It is important to note that soil factors are related here to the recruitment of bacterial taxa to the hyphae, where the physical/chemical factors probably differ from those in the soil. Finally, the present study was interested in determining whether the factors affecting soil and hyphosphere bacterial communities were similar. The Mantel test was applied to look for correlations between distance matrices for the soil versus hyphosphere communities as affected by the different fertilizer amendments. These distances were found to be linearly related for the two communities. Hence, the above-mentioned soil factors seemed to be of major importance in influencing the composition of both soil and hyphosphere communities, indicating that changes in fungal physiology between fertilizer treatments played a very minor role in influencing the hyphosphere communities. In the current soil microcosm, a mix of viable and nonviable hyphae of *P. canescens* was found, which complicates any comparison with previous studies showing a differential attachment of selected bacteria to AMF hyphae under pure-culture conditions (33).

Long-term fertilizer treatment with manure and P led to a high relative abundance of hyphosphere *Bacteroidetes*, and the phylum was even distinctive for this treatment. *Bacteroidetes* is a dominant phylum in manure (34) and is regarded as a copiotroph group that is commonly detected in C-enriched environments such as the rhizosphere (35, 36). The observed positive correlation of *Bacteroidetes* with pH was even in accordance with previous reports (31). For this PeM_1_P_1_-F community, the presence of both aerobic (Cytophagia and *Flavobacteriia*) and anaerobic taxa within the *Bacteroidetes* (*Saprospirae* and faculative anaerobic *Sphingobacteriia*) might indicate varied oxygen availability close to the hyphae.

Both *Betaproteobacteria* and *Gammaproteobacteria*, which were enriched in hyphosphere communities, respond to hyphal exudates (37) and are commonly associated with fungal hyphae (15, 16, 29). Furthermore, both taxa contain species that are rapid colonizers and can grow at interphases between aerobic and anaerobic areas (38, 39).

### Effects of fertilizer regime on bacteria community potential for phosphorus and nitrogen cycling.

Significantly increased relative abundances of several P and N cycling genes were observed in bacterial communities associated with *Penicillium* hyphae compared to those in soil communities. Additionally, fertilization treatments showed a strong impact on these gene abundances in hyphosphere communities.

*Penicillium* hyphae recruited bacteria harboring the *phoD* gene. A comparable recruitment of phosphatase-producing bacteria by hyphae of AMF has previously been reported (6). The relative abundance of *phoD* is reported to be inversely related to the level of available P in the soil and rhizosphere (40, 41). Hence, the current higher relative abundance of *phoD* in hyphosphere communities from the N_1_K_1_-F soil than in those recruited from the long-term P fertilizer-amended soils indicated that P limitation around the fungal hyphae can be relieved by long-term P application.

The high relative abundance of *phnK* in hyphosphere communities suggests that the hyphosphere bacteria utilize phosphonate. This result supports the findings for bacteria associated with *P. canescens* hyphae in a collection of very different soils (16). The phosphonates might originate from the fungal hyphae since phosphonates are reported to be components of fungal exopolysaccharides, glycoproteins, and membrane phosphonolipids (42). Phosphonates are very stable organic compounds, and bacterial phosphonate utilization has been reported in particular in phosphate-limited marine waters where the carbon-phosphorus lyase pathway for phosphonate degradation is enriched (43). It can therefore be speculated that the high relative abundance of *phnK* in most hyphosphere communities may reflect a limitation of easily available P.

The N-cycling potential of hyphosphere bacterial communities was assessed through the relative abundances of genes involved in N fixation, denitrification, and nitrification. Associations between N-fixing bacteria and fungi have previously been found and are thought to be beneficial for meeting the N requirements of fungi (1, 5). A significantly increased abundance of *nifH* in PeN_1_K_1_-F hyphosphere communities was observed, indicating recruitment of the N-fixing bacteria. This may be due to a high C/N ratio caused by fungal exudates, as seen for N fixers in the plant rhizosphere (44). The high *nifH* gene abundance might even be related to P limitation around the fungal hyphae, as N fixers have advantages in P acquisition compared to that with non-N fixers when N availability is limited (45, 46).

Higher abundances of the *nirS*, *nirK*, and *nosZ* genes, involved in denitrification, were generally observed for hyphosphere bacterial communities than for soil communities but with differences between fertilizer treatments. Interestingly, both *nirS* and *nosZ* were significantly increased in the PeN_1_K_1_-F community, whereas *nirK* was enriched in the PeM_1_P_1_-F community. For the dominating taxa in these communities, *Gammaproteobacteria* mainly harbor *nirS nosZ* genotypes, whereas *Betaproteobacteria* harbor both the *nirS nosZ* and the *nirK nosZ* genotypes (47), but the majority of *nirK*-harboring organisms do not have the *nosZ* gene (47). Overall, the distinct distributions of *nirS* and *nirK* genes across hyphosphere communities could be explained by different ecological niches of *nirS*- and *nirK*-harboring denitrifiers (48, 49).

According to previous studies, the C/N ratio is an important factor influencing denitrification, as denitrifier abundance responds positively to increased C availability in the environment (50–52). A C-rich environment around fungal hyphae due to the release of fungal exudates (37, 53, 54) might lead to the increased abundance of denitrification-related genes in this environment. Furthermore, the higher potential for denitrification could be explained partly by an anoxic microenvironment near fungal hyphae caused by the oxygen consumption of metabolically active microorganisms.

The *amoA* gene, involved in nitrification, was not enriched in hyphosphere bacterial communities. This is compatible with the evidence of a partly anoxic microenvironment near fungal hyphae discussed above, although the cooccurrence of genes for nitrification and denitrification suggests spatial or temporal heterogeneity of oxygen availability. Additionally, fungi preferentially utilize easily assimilated N sources, such as ammonium, rather than other N sources, such as nitrate, urea, and purines (55–57). Competition between fungi and bacteria could lead to a lack of ammonium around hyphae and thereby further limit nitrification.

### Conclusions.

*P. canescens* hyphae in soil recruited distinct bacterial communities dominated by *Betaproteobacteria* and *Gammaproteobacteria*, as well as by *Bacteroidetes* in soils that had been amended with manure over a long period. There were clear indications that field amendment with organic fertilizers exerted larger effects on soil and hyphosphere communities than those with amendments with mineral fertilizers. Furthermore, the potential of the hyphosphere communities for P and N cycling was generally higher than that of soil communities and was even affected by soil fertilization. The recruitment of bacteria with a potential to mobilize organic P and to fix N to the *P. canescens* hyphosphere in soils that had not received P fertilizer may be relevant for future optimization of next-generation biofertilizer consortia.

## MATERIALS AND METHODS

### Fungal strain and soil samples.

The fungus used in this study was the P-solubilizing soil isolate *Penicillium canescens* strain ATCC 10419. Three soils subjected to different fertilizer treatments were used to establish baiting soil microcosms. Soil samples were collected from the long-term nutrient depletion trial (LTNDT) established in 1964 at the experimental farm of the University of Copenhagen in Taastrup, Denmark (55°40′N, 12°17′E). Permanent mineral fertilizer (combinations of N, P, and K) and organic slurry treatments were introduced in 1995 and 1996 (17). The N_1_K_1_-F, N_1_P_2_K_2_-F, and M_1_P_1_-F soils used in this study originate from the long-term field trial. N_1_K_1_-F and N_1_P_2_K_2_-F soils received mineral fertilizers, while the M_1_P_1_-F soil received mineral P plus cattle or pig slurry, depending on the year.

### Soil microcosm setup and sample collection.

Before setting up the soil microcosms, the water content of all soil samples was adjusted to 14.3%, corresponding to ca. 50% of their water-holding capacities. These rather dry conditions were selected to avoid passive transport of bacteria from the soil to the hyphae by flowing water. All soil samples were incubated at 26°C for 8 days to activate the soil microbiota. The baiting soil microcosms were set up as in reference 15 and incubated at 26°C, the optimal growth temperature for *P. canescens*. Briefly, *P. canescens* hyphae growing on glass coverslips were placed inside polyamide mesh bags (mesh diameter, 50 μm; Sintab Produkt AB, Oxie, Sweden) and buried in the soil. There were three treatments corresponding to the three soil types, N_1_K_1_-F, N_1_P_2_K_2_-F, and M_1_P_1_-F, and each treatment included three replicates. Glass coverslips were recovered from the microcosms after incubation for up to 8 days. The coverslips were washed with sterile water to remove nonattached bacteria. From each treatment, 30 coverslips (three replicates with 10 slides per replicate) were used for DNA extractions. The hyphae and their associated bacteria were harvested by scraping off the hyphae with a sterile scalpel. For microscopic assessments, two coverslips were used for the determination of bacterial colonization. Sterile glass coverslips without fungal hyphae served as negative controls for both DNA extraction and microscopy. Triplicate soil microcosms without introduced mesh bags were used for soil DNA extraction. Detailed descriptions of the microcosm setup are presented in the supplemental material.

### Hypha viability check and bacterial colonization.

Calcofluor white M2R (CFW) and FUN1 from the LIVE/DEAD yeast viability kit (Thermo Fisher Scientific, Waltham, CA, USA) were used for staining to check the structure and viability, respectively, of hyphae on the glass coverslips at days 0, 1, 4, and 8 during incubation in the soil. SYBR green (Invitrogen Life Technologies, Carlsbad, CA, USA) staining was applied to check bacterial colonization of *P. canescens* hyphae after 8 days of incubation. The staining procedures are described in the supplemental material.

Microscopic observations of hyphae or bacteria stained with CFW, FUN1, or SYBR green were carried out using an Axioskop 2 fluorescence microscope (Zeiss, Oberkochen, Germany). A fluorescein isothiocyanate (FITC) filter (exciter filter band pass [BP], 450 to 490 nm; emission long pass [LP], 420 nm) was used to visualize fungi or bacteria stained by FUN1 and SYBR green, while a 4′,6-diamidino-2-phenylindole (DAPI) filter (exciter filter G, 365 nm; emission LP, 420 nm) was used to visualize CFW-stained hyphae or spores. All of the observations were performed using a 63× oil lens objective (total magnification, ×630; Zeiss). Images were captured using a Zeiss AxioCam digital camera and the AxioVision software.

### DNA extraction and 16S rRNA gene sequencing.

DNA was extracted from 0.25-g soil samples obtained from microcosms without the fungal inoculant using the DNeasy PowerSoil kit (Qiagen, Santa Clarita, CA, USA), according to the manufacturer’s instructions. To avoid DNA loss associated with procedures for humic acid removal inherent in the PowerSoil kit procedure, bacterial DNA was extracted from the low-biomass suspensions of hyphal and hyphosphere bacteria using the DNeasy blood and tissue kit (Qiagen). Extractions were made according to the bacterial DNA extraction protocol, including a lysozyme pretreatment, to enable DNA recovery from both Gram-negative and Gram-positive bacteria. The V3-V4 variable region of the 16S rRNA gene was amplified from DNA samples by PCR using the primer pair Bakt_341F (CCTACGGGNGGCWGCAG) and Bakt_805R (GACTACHVGGGTATCTAATCC) (58) and sequenced by the Illumina MiSeq platform (paired-end, 2 × 301 bp; Macrogen, Seoul, South Korea).

### Quantification of functional genes involved in P and N cycling.

Genes involved in P cycling (*phoD*, *phnK*, and *pqqC*) and N cycling (*nifH*, *nosZ*, *nir*S, *nirK*, and *amoA*), as well as the 16S rRNA gene, were quantified by qPCR using the Mx3000P qPCR system (Agilent Technologies, Santa Clara, CA, USA). The primers used and annealing temperatures are presented in Table 2. Twenty-microliter reaction mixtures were prepared with 10 μl of the Brilliant III Ultra-Fast SYBR green low ROX qPCR master mix (Agilent Technologies, Santa Clara, CA, USA), 1 mg/ml bovine serum albumin (BSA; Thermo Fisher Scientific, Waltham, MA), 0.4 μM each primer, and 2 μl of template DNA (1 to 10 ng/μl). Thermal cycling conditions were as follows: an initial cycle of 95°C for 3 min, followed by 40 cycles of 95°C for 20 s, and then the annealing temperature for 30 s. A dissociation curve was generated at the end of the qPCR program to verify a specific melting temperature of the amplified product. Absolute abundance was calculated based on an external standard curve for each target gene. The relative abundances of P and N cycling genes were calculated after normalization by 16S rRNA gene copy numbers, i.e., functional gene copy number/16S rRNA gene copy number. A detailed description of the cloning and validation of the standards, as well as calculation of the relative abundances of the target genes, can be found in the supplemental material.

**TABLE 2:** Primers used in this study

Target gene	Primer	Sequence	Annealing temp (°C)	Reference
16S rRNA gene	341F	CCTAYGGGRBGCASCAG	58	67
	806R	GGACTACNNGGGTATCTAAT		
*phoD*	ALPS-F730	CAGTGGGACGACCACGAGGT	58	68
	ALPS-R1101	GAGGCCGATCGGCATGTCG		
*pqqC*	pqqC2-F	AACCGCTTCTACTACCAG	58	69
	pqqC2-R	GCGAACAGCTCGGTCAG		
*phnK*	PhnK-F	CATCGTCGGCGAATCCGG	58	70
	PhnK-R	TGCTGCATGCCGCCGGAAAA		
*nifH*	nifHF	AAAGGYGGWATCGGYAARTCCACCAC	56	70
	nifHRb	TGSGCYTTGTCYTCRCGGATBGGCAT		
*nirS-1*	nirS cd3AF	GTSAACGTSAAGGARACSGG	56	70
	nirS R3cd	GASTTCGGRTGSGTCTTGA		
*nirK*	nirK-F1aCu	ATCATGGTSCTGCCGCG	58	70
	nirK-R3Cu	GCCTCGATCAGRTTGTGGTT		
*nosZ*	nosZ-F	CGYTGTTCMTCGACAGCCAG	63	71
	nosZ-1622R	CGSACCTTSTTGCCSTYGCG		72
*amoA*	amoA-1Fmod	CTGGGGTTTCTACTGGTGGTC	58	73
	GenAOBR-1	GCAGTGATCATCCAGTTGCG		

### Bioinformatics and statistical analysis.

The paired-end Illumina MiSeq reads were joined using quality score 0.9 in PANDAseq (59), generating 1,560,801 merged sequences with 464 ± 2 bp. The merged reads were further analyzed using the QIIME data analysis package (60). The QIIME pipeline included *de novo* operational taxonomic unit (OTU) picking based on 97% sequence similarity by UCLUST, representative sequence picking, representative sequence alignment, taxonomic assignment against Greengenes 13_8 (UCLUST), and tree-building steps. The resulting OTU table was filtered to remove taxa identified as chloroplasts and mitochondria. A total of 78,478 OTUs were obtained for 18 communities (nine soil samples and nine hyphosphere samples) and used in all subsequent analyses.

The alpha-diversity analyses of richness and diversity were based on data rarefied to an even depth of 34,410 reads. The richness and Shannon diversity indices were calculated via PAST 3.20 and the phyloseq package in R, respectively (61, 62). Beta diversity was analyzed by nonmetric multidimensional scaling (NMDS), and permutational multivariate analysis of variance (PERMANOVA) ordinations (10,000 permutations) was performed based on a weighted UniFrac distance matrix calculated from OTU tables using the vegan package in R (63) (https://cran.r-project.org/package=vegan).

Redundancy analysis (RDA) was conducted using the vegan package in R to explain the contribution of soil factors to changes in soil and hyphosphere bacterial communities analyzed at the phylum level. The soil factors tested were total K, exchangeable K, exchangeable Mg, total P, Olsen P, pH, and the C/N ratio. The significance of these factors was determined by the Monte Carlo permutation test (999 permutations) in the vegan package. The Mantel test was used to determine the correlation between soil and hyphosphere bacterial communities. This analysis was carried out for soil and hyphosphere bacterial community distance matrices (unweighted UniFrac and weighted UniFrac) in QIIME (http://qiime.org/scripts/compare_distance_matrices.html). The results contain the Mantel *r* statistic and *P* value; *r* refers to the correlation which falls in the range of −1 (negative correlation) to +1 (positive correlation). A two-tailed test was performed to calculate the *P* value (999 permutations).

Hierarchical cluster analysis was performed based on Bray-Curtis dissimilarity matrices of relative abundances of bacterial OTUs at the phylum and class levels. The linear discriminant analysis effect size (LEfSe) algorithm was performed using the Kruskal-Wallis test and pairwise Wilcoxon test (64) (https://huttenhower.sph.harvard.edu/galaxy). Linear discriminant analysis was used to estimate the eﬀect size of each differentially abundant taxon. An alpha significance level of 0.5 and an effect size threshold of 2.0 were used for all distinctive taxa. Statistical analyses of enriched OTUs and ternary plots were performed using the R package edgeR (65) with scripts from https://www.mpipz.mpg.de/R_scripts (66). Analyses included OTUs with relative abundances above 0.05%. Briefly, a negative binomial generalized linear model (GLM) was fitted to the OTUs with relative abundances above 0.05% for selecting OTUs with differential abundances among the compared samples. Phylum-level classification of each enriched OTU was obtained, and the relative abundances of enriched OTUs were visualized with heat maps after centering and scaling by the pheatmap package in R.

The significance of differences in alpha-diversity indices and relative abundances of functional genes were determined by one-way analysis of variance (ANOVA), followed by the *post hoc* Duncan test and by Tukey’s honestly significant difference (HSD) test using the SPSS software version 19 (Armonk, NY, USA).

### Data availability.

Raw sequence data were submitted to the NCBI Sequence Read Archive and assigned accession number SRP179700.

## Supplementary Material

Supplemental file 1
