# Supplementary material for: Different Effects of Soil Fertilization on Bacterial Community Composition in the Penicillium canescens Hyphosphere and in Bulk Soil
Source: Appl Environ Microbiol. 2020 May 5;86(10):e02969-19. doi: 10.1128/AEM.02969-19 (PMC7205497; doi:10.1128/AEM.02969-19)
Supplement: Supplemental file 1 [file AEM.02969-19-s0001.pdf]

Supplementary material

Materials and Methods

Setup of baiting microcosm systems and collection of samples

200 µl of *Penicillium canescens* spore suspension (1 × 10^6 spores/ml) were incubated on 1/5 strength Potato Dextrose Agar (PDA, Difco Laboratories) at 26 ˚C for one day. Fungal plugs from the PDA plates were transferred to sterile glass cover slips that were subsequently placed on 1.5% water agar plates and incubated at 26 ˚C for 3 days. After that, fungal plugs were removed and the glass slips covered by hyphae were put into sterilized polyamide mesh bags (Sintab Produkt AB, 50 µm mesh diameter). The mesh bags with glass slips were buried in soil microcosms, which consisted of petri dishes filled with 100 g of soil, ensuring a good contact of mesh bags and their enclosed cover slips with the soil. Petri dishes were sealed with Parafilm to reduce the loss of water. After 8 days of incubation at 26 ˚C, glass slips were taken out from the microcosms and gently washed twice with 500 µl of sterile Milli-Q water to remove bacteria, which were not associated with hyphae (1). Subsequently, 200 µl of sterile Milli-Q water were added on each glass slip and the hyphae plus bacteria colonizing hyphae were scraped off with a sterile scalpel. The suspension was transferred to a sterile tube. The bacteria and hyphae from ten glass slips were collected in one tube as one replicate. Each treatment had three replicates and all samples were stored at -80 ˚C before DNA extraction.

Calcofluor® White M2R (CFW) and FUN® 1 Staining

To demonstrate presence and viability of *Penicillium canescens* hyphae introduced into soil microcosms, glass slips covered with hyphae were collected from soil microcosms at different time points (day 0, day 1, day 4 and day 8). N_1P_1-F soil was chosen for hyphae viability check. CFW bind to chitin of fungal cell-walls and cause them to exhibit blue fluorescence (2). To stain chitin of cell walls, 30 µl of 25 µM CFW was incubated with hyphae on glass slips at room temperature in dark for 10 minutes. FUN® 1 is a two-color fluorescent viability probe, which determines the metabolic activity of fungal cells (3). For viability staining, 30 µl of 25 µm FUN 1 and hyphae were incubated at 30 ˚C in dark for 1.5 hours before checking metabolic state of the hyphae. The active hyphae or spores exhibited a striking red-orange fluorescence, while dead hyphae and spores were stained with a diffusely distributed green fluorescence. Each staining process used at least two glass slips.

To determine colonization of *P. canescens* hyphae by bacteria, glass slips covered by hyphae were collected from soil microcosms at day 8, and 20 µl SYBR Green (1:100 dilution from stock) was added to stain hyphae, spores and bacteria. After co-incubation at room temperature for 5 minutes, stained fungi and bacteria were observed using an epifluorescence microscope.

Quantification of functional genes involved in P and N cycling

The Phosphorus cycling genes target by the current qPCR analysis included *phoD* encoding alkaline phosphatase, *phnK* which is involved in phosphonate utilization and *pqqC* involved in pyrroloquinoline quinone biosynthesis associated with inorganic P solubilization. The target functional genes involved in nitrogen metabolism were *nifH* (Nitrogenase iron protein), *nosZ* (Nitrous-oxide reductase), *nir*S (Nitrite reductase), *nirK* (Nitrite reductase) and *amoA* (ammonia monooxygenase). Before running qPCR, reactions, reference standards of functional genes and 16S rRNA genes were prepared. Initially, the 16S rRNA gene and genes related to P and N cycling were amplified from the bulk soil using specific primers (Table S2). PCR reactions (50 µl) had final concentrations of 3 µM $MgCl_2$, 0.5 µM dNTP, 0.2 µM of each primer, 0.05 U/µl Taq DNA polymerase (Sigma-Aldrich, St. Louis, MO, USA), 1×PCR buffer without $MgCl_2$ provided with the DNA polymerase, and 2 µl of DNA template. Thermal cycling was initiated by heating to 95°C for 5 min, followed by 35 cycles of 95°C for 30 s, annealing temperature (see Table S2) for 30 s, and 72°C for 30 s, and a final extension at 72°C for 1 min. Subsequently, standard plasmids carrying insertions of amplified target genes were constructed using the TOPO® TA Cloning® Kit (Invitrogen, Carlsbad, CA, USA). PCR amplicons were purified by the QIAquick Gel Extraction Kit (Qiagen, Santa Clarita, CA, USA), and then cloned into competent TOP10 *E. coli* cells using the TOPO® TA Cloning® Kit (Invitrogen) with the pCR™2.1-TOPO® vector according to the manufacturer's instructions. The colonies containing plasmids with correct insertion were chosen based on colony PCR results. After that, plasmids were extracted and purified by the QIAprep Miniprep Kit (Qiagen) and sequenced (GATC Biotech, Germany) to verify the identity of the inserts. These standard plasmids were used to prepare standard curves for the respective qPCR analyses.

Copy numbers of target genes were calculated according to the plasmid DNA concentration as measured by Nanodrop, using the equation as follows:

$$\text{Target gene (copies/µl)} = \frac{6.02 \times 10^{14} \times \text{Plasmid DNA concentration (ng/µl)}}{660 \times (\text{length of vector} + \text{length of target gene})}.$$

Then the relative abundance of specific functional genes normalized to the 16S rRNA gene (copy numbers/ 16S rRNA gene) was calculated.

References

1. Ghodsalavi B, Svenningsen NB, Hao X, Olsson S, Nicolaisen MH, Al-Soud WA, Sørensen SJ, Nybroe O. 2017. A novel baiting microcosm approach used to identify the bacterial community associated with *Penicillium bilaii* hyphae in soil. PloS one 12: e0187116.

2. Hughes J, McCully ME. 1975. The use of an optical brightener in the study of plant structure. Stain Technol 50: 319-329.

3. Millard PJ, Roth BL, Thi HP, Yue ST, Haugland RP. 1997. Development of the FUN-1 family of fluorescent probes for vacuole labeling and viability testing of yeasts. Appl Environ Microbiol 63: 2897–2905.

Table S1 P-values of Student’s t- test comparing relative abundances of major bacterial phyla between soil and hyphosphere communities.

Phylum (Relative Abundance >2%)	Student's t-test		
	PeN_1K_1- F	$PeN_1P_2K_2$-F	PeM_1P_1-F
Proteobacteria	0.064	0.000	0.002
Actinobacteria	0.001	0.001	0.002
Verrucomicrobia	0.000	0.003	0.001
Firmicutes	0.099	0.001	0.030
Bacteroidetes	0.526	0.001	0.003
Gemmatimonadetes	0.002	0.002	0.002
Planctomycetes	0.002	0.001	0.001
Acidobacteria	0.004	0.002	0.001

P-values in green mean that bacterial relative abundances were significantly higher in hyphosphere samples than in corresponding bulk soil samples; P-values in red refer to the opposite case.

Table S2 Distinctive bacterial taxa of hyphosphere communities depending on different soil background.

Distinctive bacterial taxa (from phylum to genus level)	Hyphosphere sample
Bacteria|Firmicutes|Bacilli|Bacillales|Paenibacillaceae	PeN_1K_1- F
Bacteria|Actinobacteria|Actinobacteria|Actinomycetales|Microbacteriaceae|Glaciibacter	PeN_1K_1- F
Bacteria|Tenericutes|Mollicutes|Anaeroplasmatales	PeN_1K_1- F
Bacteria|Firmicutes|Bacilli|Bacillales|Paenibacillaceae|Paenibacillus	PeN_1K_1- F
Bacteria|Firmicutes|Bacilli|Bacillales|Staphylococcaceae|Staphylococcus	PeN_1K_1- F
Bacteria|Firmicutes|Bacilli|Bacillales|Staphylococcaceae	PeN_1K_1- F
Bacteria|Tenericutes|Mollicutes|Anaeroplasmatales|Anaeroplasmataceae	PeN_1K_1- F
Bacteria|Tenericutes|Mollicutes	PeN_1K_1- F
Bacteria|Tenericutes	PeN_1K_1- F
Bacteria|Actinobacteria|Actinobacteria|Actinomycetales|Propionibacteriaceae|Propionibacterium	PeN_1K_1- F
Bacteria|Actinobacteria|Actinobacteria|Actinomycetales|Propionibacteriaceae	PeN_1K_1- F
Bacteria|Proteobacteria|Betaproteobacteria|Neisseriales|Neisseriaceae	PeN_1K_1- F
Bacteria|Proteobacteria|Betaproteobacteria|Neisseriales|Neisseriaceae|Microvirgula	PeN_1K_1- F
Bacteria|Firmicutes|Bacilli|Bacillales|Planococcaceae|Sporosarcina	PeN_1K_1- F
Bacteria|Actinobacteria|Thermoleophilia|Solirubrobacterales|Patulibacteraceae	$PeN_1P_2K_2$-F
Bacteria|Proteobacteria|Gammaproteobacteria|Xanthomonadales|Xanthomonadaceae|Luteibacter	$PeN_1P_2K_2$-F
Bacteria|Proteobacteria|Gammaproteobacteria|Xanthomonadales|Xanthomonadaceae	$PeN_1P_2K_2$-F
Bacteria|Proteobacteria|Gammaproteobacteria|Xanthomonadales	$PeN_1P_2K_2$-F
Bacteria|Actinobacteria|Actinobacteria|Actinomycetales|Microbacteriaceae|Cryobacterium	$PeN_1P_2K_2$-F
Bacteria|Proteobacteria|Betaproteobacteria|Burkholderiales|Burkholderiaceae|Salinispora	$PeN_1P_2K_2$-F
Bacteria|Proteobacteria|Alphaproteobacteria|Rhizobiales	PeM_1P_1-F
Bacteria|Actinobacteria|Actinobacteria|Actinomycetales|Actinosynnemataceae|Lentzea	PeM_1P_1-F
Bacteria|Actinobacteria|Actinobacteria|Actinomycetales|Actinosynnemataceae	PeM_1P_1-F

Bacteria|Proteobacteria|Gammaproteobacteria|Xanthomonadales|Xanthomonadaceae|Arenimonas	PeM_1P_1-F
Bacteria|Bacteroidetes|Flavobacteriia|Flavobacteriales|_Weeksellaceae_	PeM_1P_1-F
Bacteria|Proteobacteria|Alphaproteobacteria|Rhizobiales|Rhizobiaceae	PeM_1P_1-F
Bacteria|Bacteroidetes|Sphingobacteriia|Sphingobacteriales|Sphingobacteriaceae	PeM_1P_1-F
Bacteria|Proteobacteria|Alphaproteobacteria	PeM_1P_1-F
Bacteria|Proteobacteria|Deltaproteobacteria|Myxococcales|Haliangiaceae	PeM_1P_1-F
Bacteria|Bacteroidetes|Flavobacteriia	PeM_1P_1-F
Bacteria|Proteobacteria|Alphaproteobacteria|Rhizobiales|Phyllobacteriaceae|Mesorhizobium	PeM_1P_1-F
Bacteria|Bacteroidetes|Cytophagia|Cytophagales	PeM_1P_1-F
Bacteria|Bacteroidetes|Cytophagia|Cytophagales|Cytophagaceae	PeM_1P_1-F
Bacteria|Actinobacteria|Actinobacteria|Actinomycetales|Actinosynnemataceae|Kibdelosporangium	PeM_1P_1-F
Bacteria|Proteobacteria|Gammaproteobacteria|Xanthomonadales|Xanthomonadaceae|Thermomonas	PeM_1P_1-F
Bacteria|Proteobacteria|Alphaproteobacteria|Rhizobiales|Rhizobiaceae|Rhizobium	PeM_1P_1-F
Bacteria|Bacteroidetes|Cytophagia	PeM_1P_1-F
Bacteria|Bacteroidetes|Flavobacteriia|Flavobacteriales	PeM_1P_1-F
Bacteria|Proteobacteria|Betaproteobacteria|Burkholderiales|Oxalobacteraceae|Cupriavidus	PeM_1P_1-F
Bacteria|Proteobacteria|Gammaproteobacteria|Xanthomonadales|Xanthomonadaceae|Lysobacter	PeM_1P_1-F
Bacteria|Bacteroidetes|Sphingobacteriia|Sphingobacteriales	PeM_1P_1-F
Bacteria|Bacteroidetes|Sphingobacteriia	PeM_1P_1-F

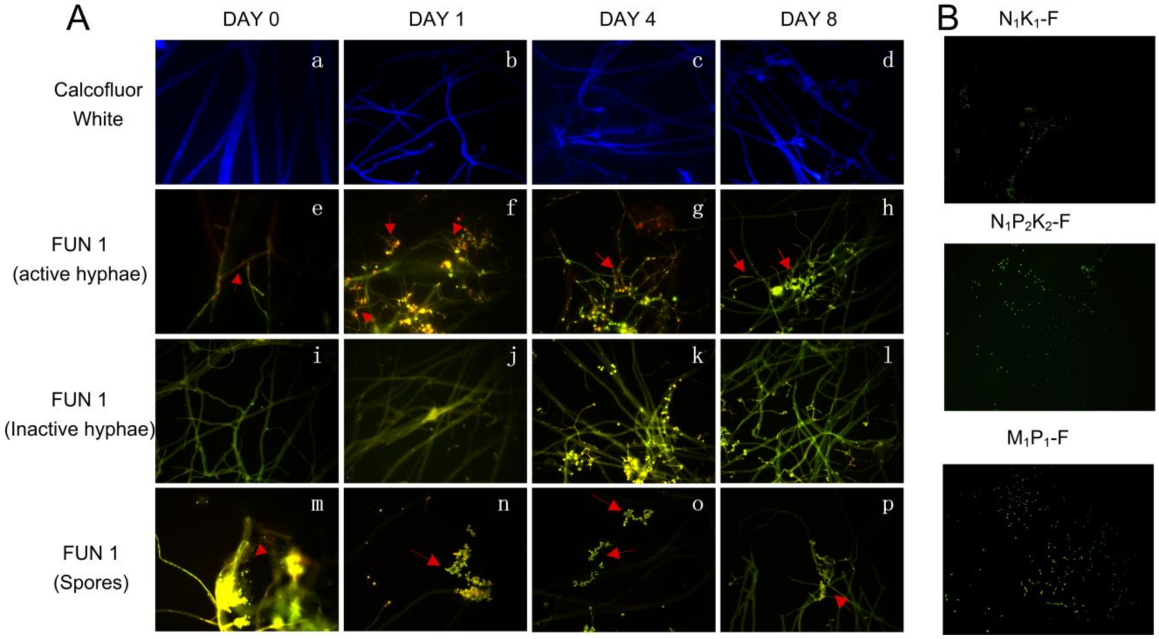

Figure S1 Structure and viability of fungal hyphae and bacterial attachment onto glass slips without *P. canescens* hyphae. **(A)** Visualization of the structure and viability of hyphae from *P. canescens*. Cover slips covered by hyphae and spores were recovered from N_1K_1-F soil microcosms at day 0, 1, 4 and 8. Images a-d show *P. canescens* hyphae stained by Calcofluor® White M2R (CFW). The metabolic states of hyphae and spores were determined by Fun 1 staining (images e-p). Metabolic active hyphae (images e-h) and spores (images m-p) exhibited red-orange fluorescence, while hyphae without metabolically activities showed diffused yellow-green-fluorescence (images i-l). Red arrows point to metabolically active hyphae or spores. **(B)** Bacterial attachment onto negative control glass slips without *P. canescens* hyphae from microcosms with N_1K_1-F, $N_1P_2K_2$-F and M_1P_1-F soils. All Images were obtained by fluorescence microscopy at 630x magnification.

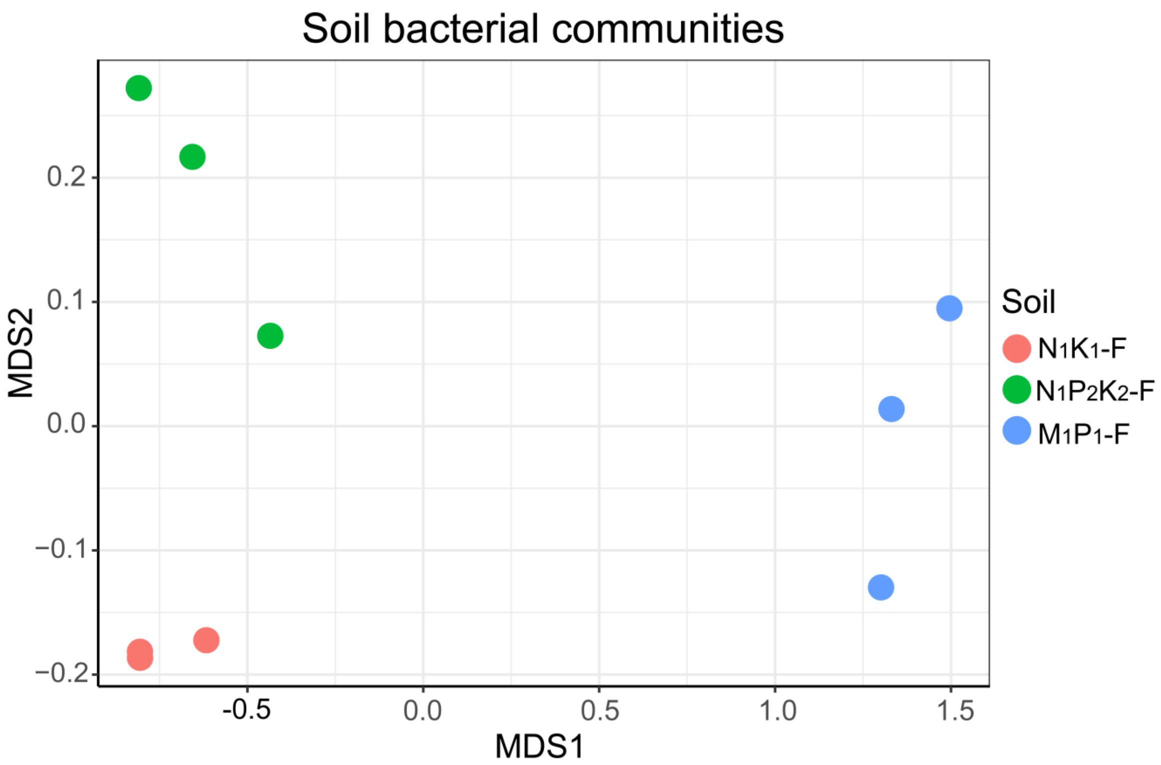

Figure S2 The non-metric multidimensional scaling (NMDS) plots for soil bacterial community structure based on weighted Unifrac distances.

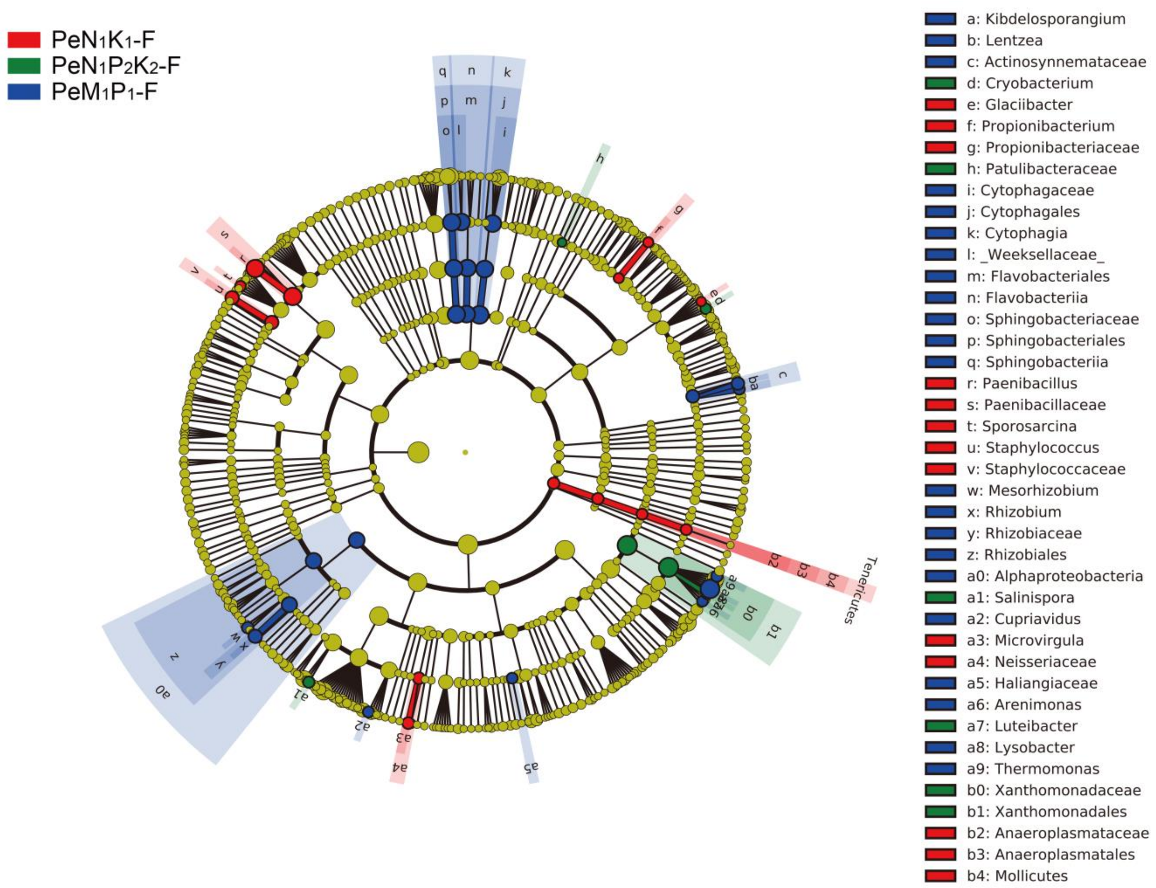

Figure S3 Distinctive taxa among the three hyphosphere samples after LEfSe analysis. Differentially abundant taxa were highlighted by nodes and shading with different colors. Yellow nodes represent taxa with no significant differences in relative abundance among different samples. Rings represent phylum, class, order, family and genus from insides to outside of the cladogram.

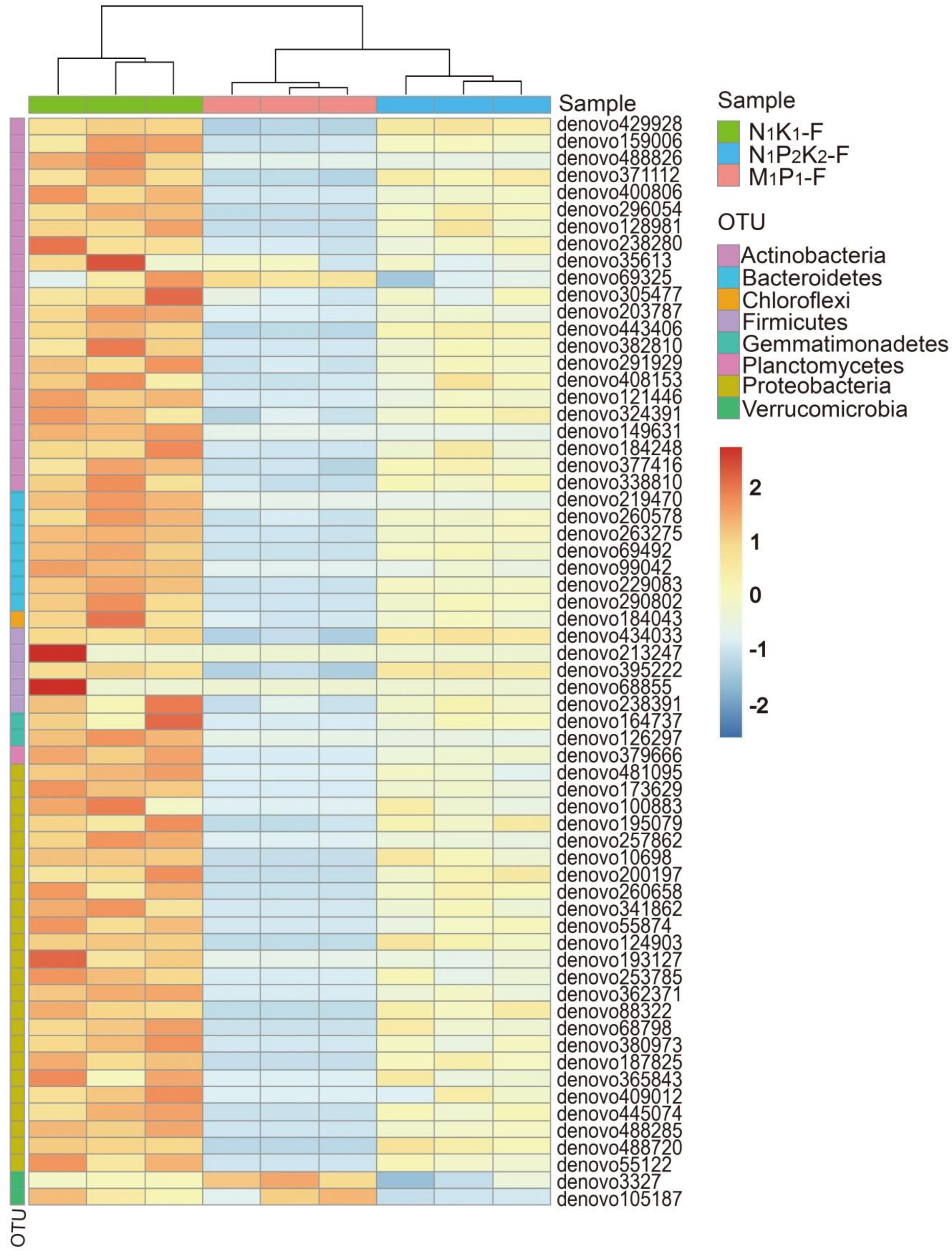

Figure S4 Heat map of the relative abundance of OTUs enriched in the N_1K_1-F soil. Abundance data were centered and scaled to each OTU's abundance by the Pheatmap package in R. Each vertical column corresponds to one replicate of a specific soil sample. The annotations on the left side of the heat map show the classification of each OTU at the phylum level.

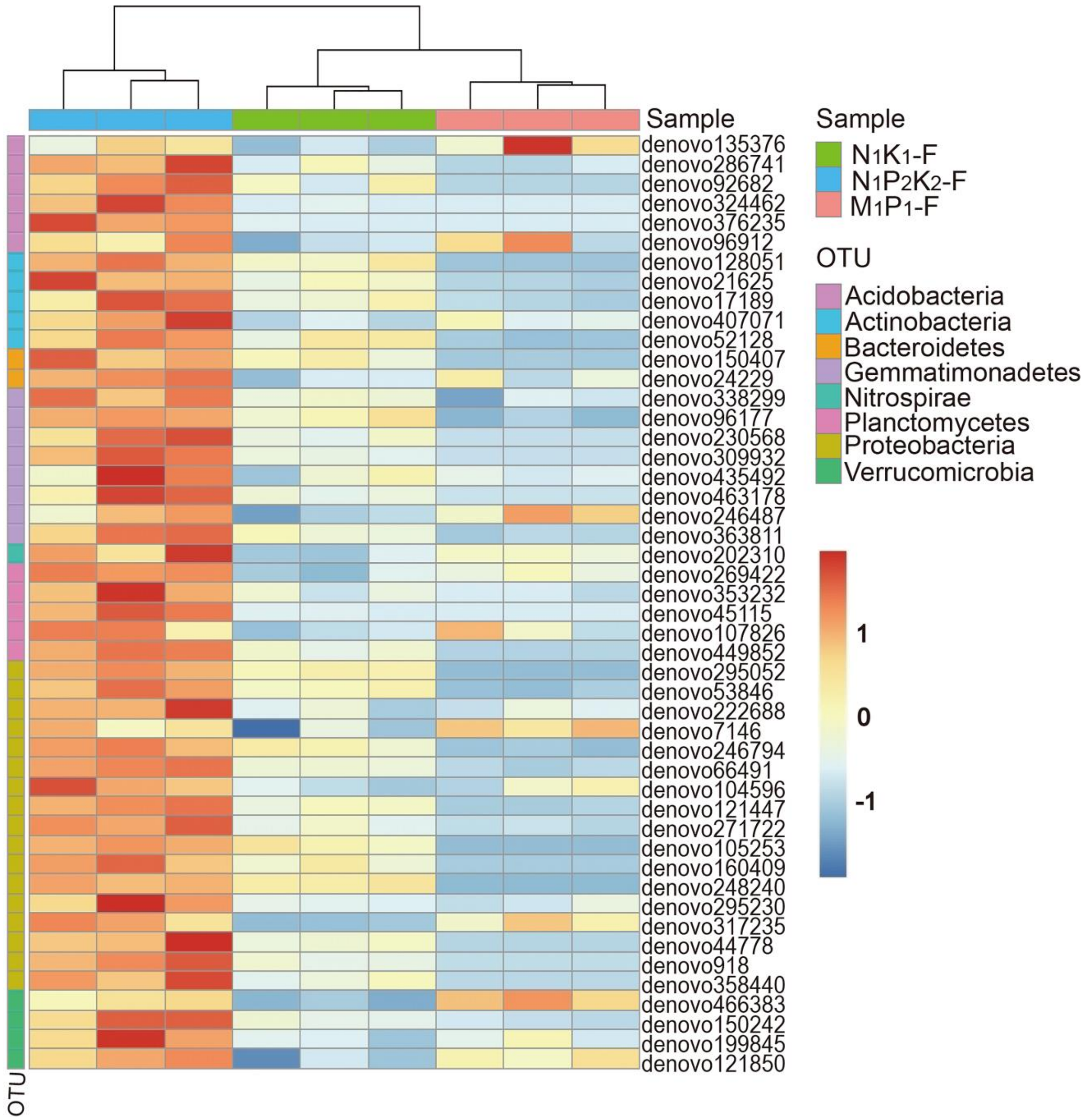

Figure S5 Heat map of the relative abundance of OTUs enriched in the $N_1P_2K_2$-F soil. Abundance data were centered and scaled to each OTU's abundance by the Pheatmap package in R. Each vertical column corresponds to one replicate of a specific soil sample. The annotations on the left side of the heat map show the classification of each OTU at the phylum level.

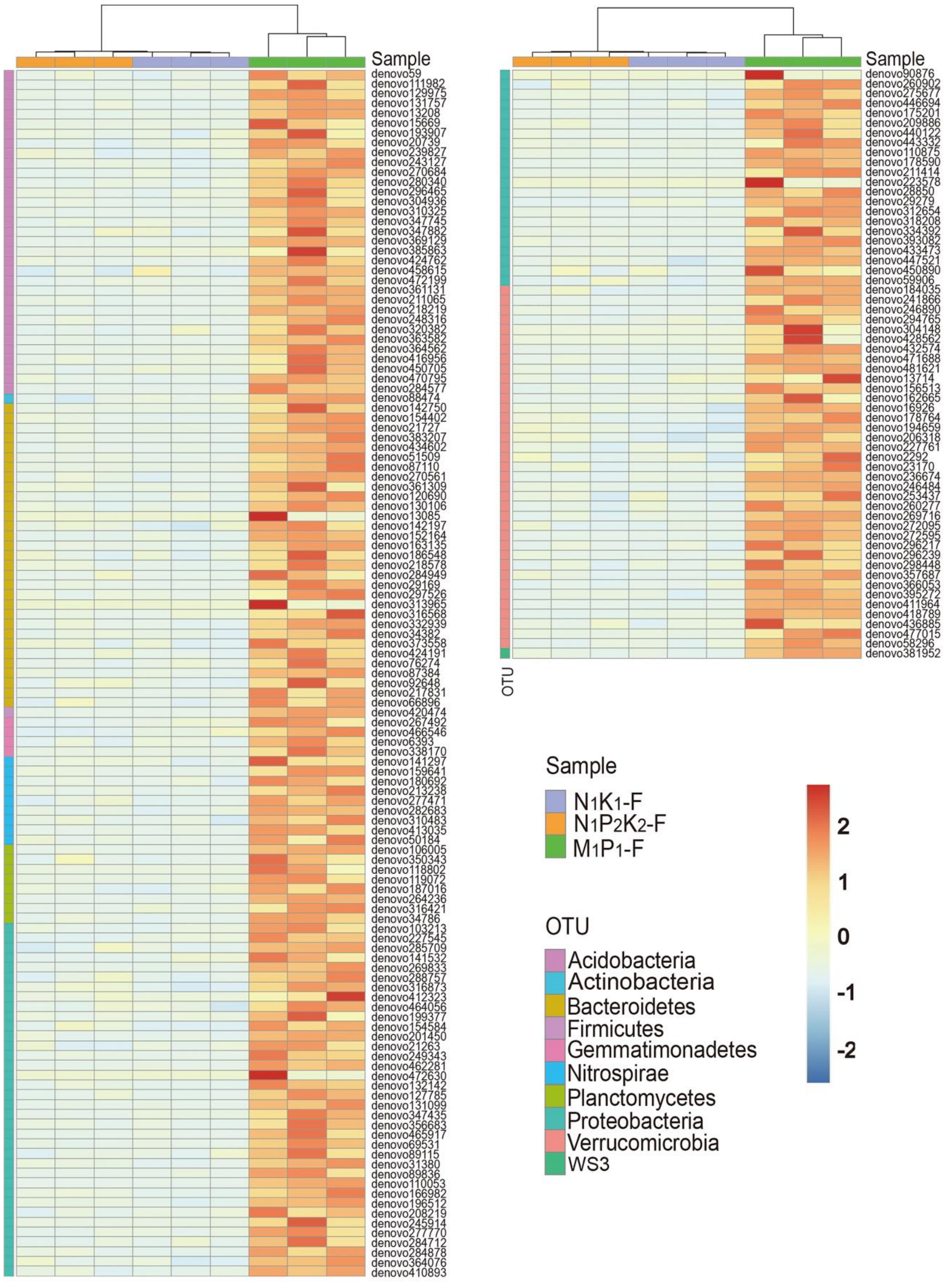

Figure S6 Heat map of the relative abundance of OTUs enriched in the M_1P_1-F soil. Abundance data were centered and scaled to each OTU's abundance by the Pheatmap package in R. Each vertical column corresponds to one replicate of a specific soil sample. The annotations on the left side of the heat map show the classification of each OTU at the phylum level.

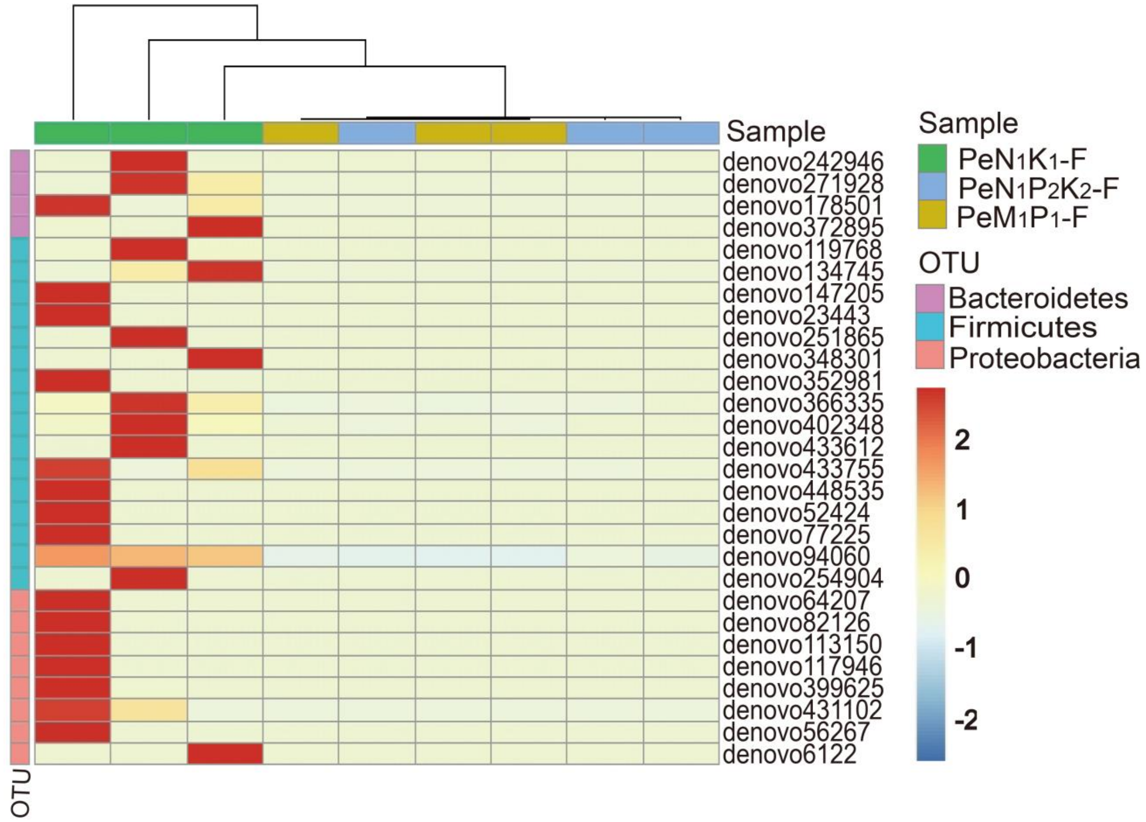

Figure S7 Heat map of the relative abundance of OTUs enriched in the PeN_1K_1-F hyphosphere. Abundance data were centered and scaled to each OTU's abundance by the Pheatmap package in R. Each vertical column corresponds to one replicate of a specific hyphosphere sample. The annotations on the left side of the heat map show the classification of each OTU at the phylum level.

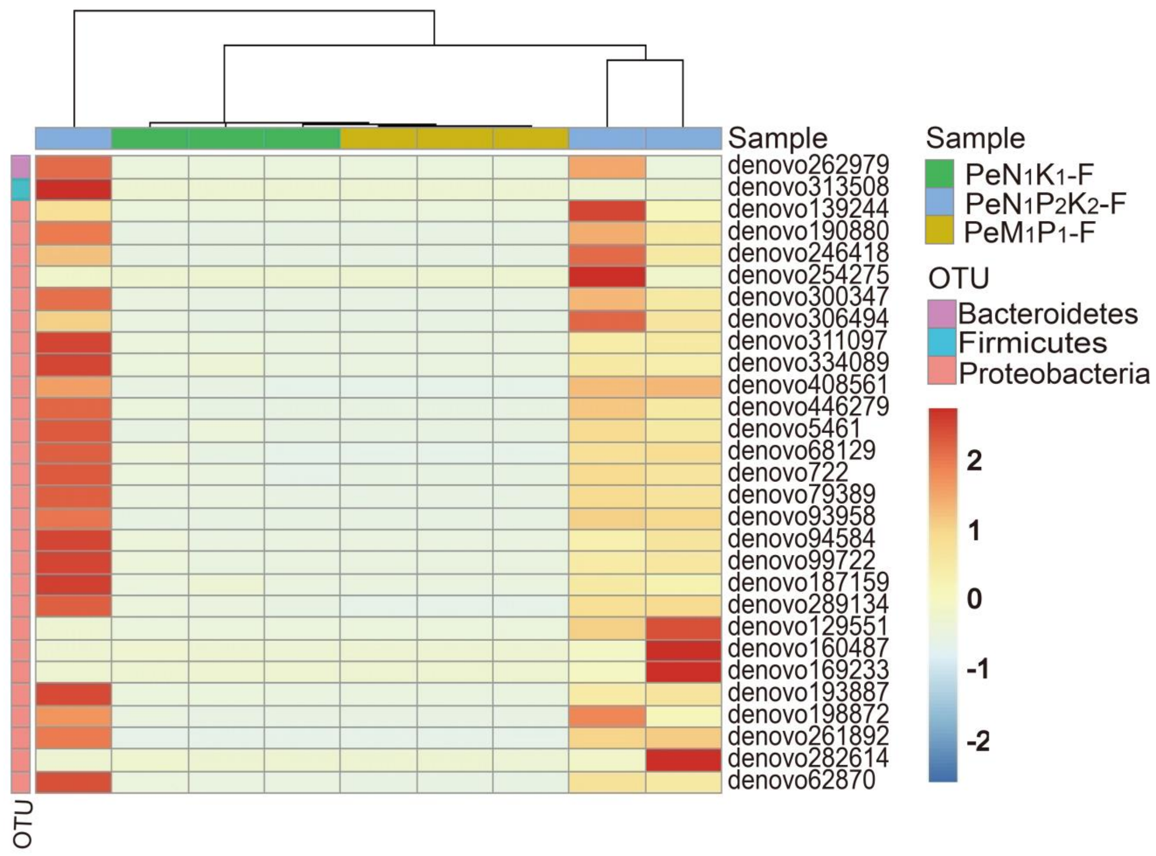

Figure S8 Heat map of the relative abundance of OTUs enriched in the $PeN_1P_2K_2$-F hyphosphere. Abundance data were centered and scaled to each OTU's abundance by the Pheatmap package in R. Each vertical column corresponds to one replicate of a specific hyphosphere sample. The annotations on the left side of the heat map show the classification of each OTU at the phylum level.

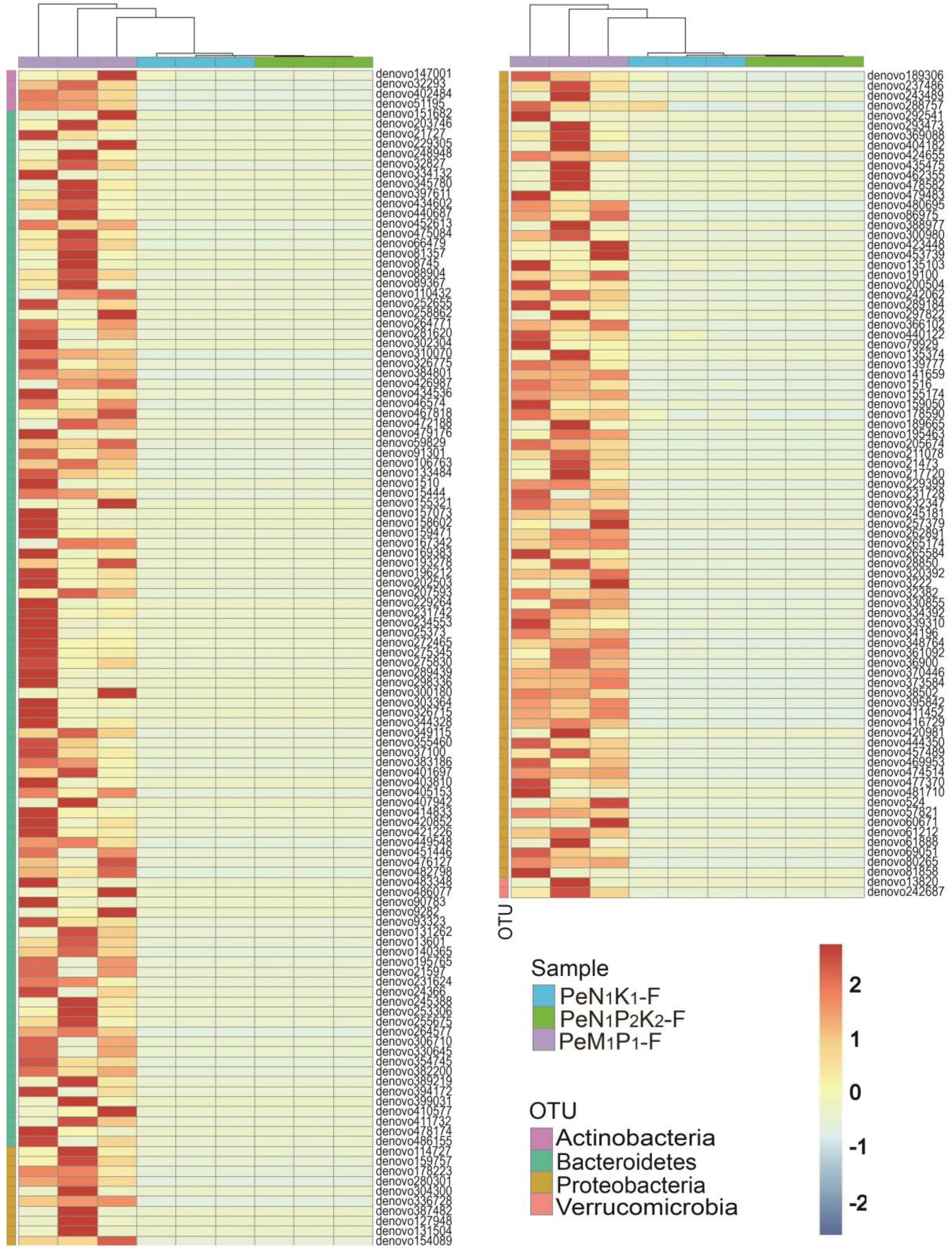

Figure S9 Heat map of the relative abundance of OTUs enriched in the PeM_1P_1-F hyphosphere. Abundance data were centered and scaled to each OTU's abundance by the Pheatmap package in R. Each vertical column corresponds to one replicate of a specific hyphosphere sample. The

annotations on the left side of the heat map show the classification of each OTU at the phylum level.
